# Enhancing Bidirectional Modbus TCP ↔ RTU Gateway Performance: A UDP Mechanism and Markov Chain Approach

**DOI:** 10.3390/s25133861

**Published:** 2025-06-21

**Authors:** Shuang Zhao, Qinghai Zhang, Qingjian Zhao, Xiaoqian Zhang, Yang Guo, Shilei Lu, Liqiang Song, Zhengxu Zhao

**Affiliations:** 1 Shandong Key Laboratory of Space Debris Monitoring and Low-Orbit Satellite Networking, Qingdao University of Technology, Qingdao 266520, China; zhangqinghai@qut.edu.cn (Q.Z.); zhaoqingjian@stu.qut.edu.cn (Q.Z.); zhangxiaoqian@stu.qut.edu.cn (X.Z.); guoyang@qut.edu.cn (Y.G.); lushilei@qut.edu.cn (S.L.); zhaozhengxu@qut.edu.cn (Z.Z.); 2 National Astronomical Observatories, Chinese Academy of Sciences, Beijing 100101, China; lqsong@bao.ac.cn

**Keywords:** IIoT, communication protocols, Modbus gateway, TCP/UDP, real-time optimization, AUDP, reliability mechanism, Markov chain

## Abstract

In the Industrial Internet of Things (IIoT) field, the diversity of devices and protocols leads to interconnection challenges. Conventional Modbus Transmission Control Protocol (TCP) to Remote Terminal Unit (RTU) gateways suffer from high overhead and latency of the TCP protocol stack. To enhance real-time communication while ensuring reliability, this study applies Markov chain theory to analyze User Datagram Protocol (UDP) transmission characteristics. An Advanced UDP (AUDP) protocol is proposed by integrating a Cyclic Redundancy Check (CRC) check mechanism, retransmission mechanism, Transaction ID matching mechanism, and exponential backoff mechanism at the UDP application layer. Based on AUDP, a Modbus AUDP-RTU gateway is designed with a lightweight architecture to achieve bidirectional conversion between Modbus AUDP and Modbus RTU. Experimental validation and Markov chain-based modeling demonstrate that the proposed gateway significantly reduces communication latency compared to Modbus TCP-RTU and exhibits higher reliability than Modbus UDP-RTU.

## 1. Introduction

In recent years, with the development of the Industrial Internet of Things (IIOT), machine-to-machine (M2M) communication technology has been widely adopted in industrial fields. However, the fragmented nature of IoT solutions compels industrial systems to integrate multi-source heterogeneous technologies for automated control, thereby urgently requiring the establishment of efficient interoperability mechanisms to ensure seamless collaboration among heterogeneous devices. Consequently, many organizations are developing new and existing IoT standards, platforms, and communication protocols to meet the demands of emerging Industry 4.0 [1]. Currently popular communication protocols include Modbus, CAN (Controller Area Network), PROFINET (Process Field Network), etc. [2]. Among these, Modbus has gained significant favor among industry organizations and equipment manufacturers due to its lightweight simplicity and ease of use [3]. With the increasing number of connected devices in industrial IoT, there is a growing demand for higher capacity in communication bus connections. The Modbus can connect up to 247 slave devices at once, surpassing most communication protocols, thus highlighting its high practical value. Therefore, developing a high-performance Modbus protocol conversion gateway is not only a technical challenge but also a practical necessity in the industrial field.

The Modbus protocol, developed by Modicon as a master–slave communication architecture [4], was initially designed to provide a lightweight solution for control data transmission between controllers and sensors via serial links. Modbus dominated industrial distributed control systems (DCSs) from the 1990s to the early 2000s. Although the industrial sector has gradually been migrating toward service-oriented protocols such as Open Platform Communications Unified Architecture (OPC UA) and Message Queuing Telemetry Transport (MQTT), Modbus continues to play a pivotal role at the field level, particularly for the large installed base of legacy PLCs and remote I/O devices that remain in operation [5]. Modbus RTU and Modbus ASCII—two serial-based variants of the protocol—enable efficient communication between sensor/actuator-level devices and supervisory control layers [6]. Schneider Electric later extended the protocol’s applicability through Modbus TCP, which supports heterogeneous network media [7], thereby enabling client–server communication across diverse bus systems and networked industrial devices. The Modbus protocol exhibits inherent limitations in practical implementations. Modbus RTU (serial-based) and Modbus TCP/UDP (Ethernet-based) demonstrate divergent performance characteristics [8]. Specifically, Modbus RTU suffers from lower data rates, constrained transmission distances, and complex cabling compared to its TCP/UDP counterparts. Conversely, Modbus TCP/UDP introduces packet loss vulnerabilities and reduced reliability relative to RTU, alongside challenges in cost and backward compatibility. Consequently, hybrid architectures integrating serial and Ethernet networks are often adopted [9]. While Modbus TCP enhances reliability through congestion control algorithms [10], this comes at the expense of increased latency—particularly pronounced under suboptimal network conditions where TCP latency significantly exceeds that of connectionless UDP [11,12,13]. However, as the demand for industrial automation continues to grow, higher demands are being placed on the real-time reliability of industrial networks [14,15]. The minimal protocol overhead of UDP eliminates handshake-induced delays [16], making it attractive for time-sensitive applications [17]. Yet its lack of built-in reliability mechanisms compromises communication integrity [18]. Furthermore, the protocol incompatibility between serial Modbus and Ethernet-based variants necessitates gateway-mediated protocol conversion to bridge these heterogeneous networks. The existing conversion methods include using visual tools such as Node-RED and hardware protocol conversion gateways to achieve interoperability of heterogeneous data. Node-RED is a programming tool that aggregates hardware devices, Application Programming Interface (API), and online services, enabling the exchange of heterogeneous data through logical orchestration. It plays a significant role in cross-platform human–machine interfaces (HMIs) and has been widely used in IoT systems [19]. However, its reliance on software stack results in higher latency compared to hardware protocol conversion gateways, leading to lower real-time performance. In some scenarios, this may fail to meet the high real-time requirements. Therefore, hardware conversion gateways still have an irreplaceable role to play.

Modbus gateways predominantly support protocol interconversion between Modbus TCP and RTU. From the above discussion, it is clear that under weak network conditions, TCP can exhibit higher latency compared to UDP and cannot accommodate certain high real-time scenarios, while UDP itself lacks reliability. Modbus gateways based on these transport protocols face the same challenges. This study presents a novel gateway design, enabling bidirectional conversion between Modbus UDP and Modbus RTU protocols. Leveraging the low-latency advantages of UDP, the proposed architecture introduces application-layer reliability mechanisms to enhance data transmission robustness, thereby achieving deterministic real-time performance while mitigating the inherent reliability limitations of UDP. The specific objectives encompass the following:

- Utilizing the connectionless nature of UDP to dramatically reduce data transfer delays on the Ethernet side of the Modbus gateway in weak network conditions.
- Adopting Markov chain model to analyze Modbus UDP transmission in depth, and proposing an improved UDP protocol, AUDP, i.e., add retransmission, data checking, and confirmation mechanism in the UDP application layer to improve the reliability of UDP transmission itself appropriately.
- Develop a Modbus AUDP and Modbus RTU protocol conversion module, design a Modbus AUDP to Modbus RTU experimental gateway, and conduct data transmission verification tests.
- Analyze and compare the test data using the Markov chain model to verify the performance of the experimental gateway, especially under weak network conditions.

The subsequent sections of this paper are organized as follows: Section 2 first introduces the current status of Modbus TCP gateways to highlight issues related to UDP applications. Then, we conduct a theoretical analysis of the real-time performance of Modbus TCP and Modbus UDP, demonstrating the necessity and feasibility of improving Modbus gateways. Finally, we briefly introduce the current applications and principles of Markov chains, which provide the theoretical foundation for the subsequent analyses in this paper, such as supporting the necessity of certain mechanisms in AUDP (Section 3.3.2) and offering theoretical support for the in-depth analysis of the final experimental results (Section 4.3). Section 3 introduces the methodology, including hardware design and software design, and the hardware design includes the design of the overall system framework and hardware architecture, and the software design mainly includes the design of the AUDP mechanism and protocol conversion with the help of Markov chain. Section 4 carries out the experimental validation and analyzes the experimental results based on the Markov chain. Section 5 summarizes the whole paper and looks forward to the direction of future research.

## 2. Related Works

### 2.1. Modbus Gateway

**Figure 1:**
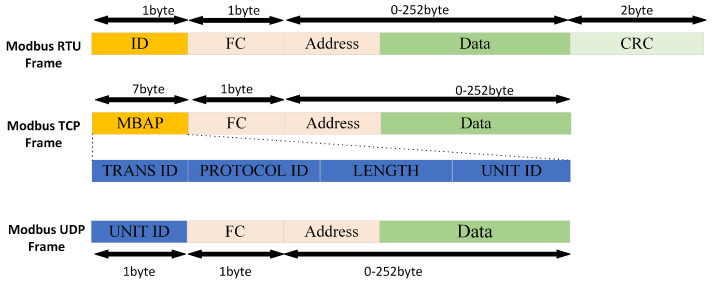
Modbus RTU, Modbus TCP, and Modbus UDP frame structure.

The frame structures of the three Modbus protocol variants are illustrated in Figure 1. While these protocols achieve transparent application-layer communication, their incompatibilities at the physical layer, data link layer (frame formats), and transport layer necessitate protocol conversion for cross-domain interoperability. Current research has predominantly focused on Modbus TCP-RTU interconversion. Sadik Tamboli et al. [20] designed a Modbus TCP-RTU gateway to communicate between the S7-1200 and the DSC and Controllogix parts of the system. Chang Cheng et al. [21] developed an STM32F107-based hardware platform with an RT-Thread real-time operating system, implementing protocol conversion between Modbus and Transmission Control Protocol/Internet Protocol (TCP/IP) to interconnect fieldbus networks with Ethernet. Abhay Sharma et al. [22] created a C library and socket programming framework for Modbus RTU-TCP/IP conversion. Adrian Korodi et al. [23] employed Node-RED as a protocol conversion middleware, where Modbus nodes were used to acquire field device data (e.g., PLC register values), followed by data processing (such as unit conversion and JSON formatting), ultimately publishing the transformed data as standardized information models via OPC UA nodes. Tao M et al. [24] designed a dual-line Ethernet protocol conversion gateway that converts Modbus TCP to MQTT protocol, transmits data to the Alibaba Cloud IoT Platform, and enables Modbus TCP data reception with MQTT communication. Sonia Palaguachi et al. [25] proposed an architecture for a hierarchical sensor network using the MQTT protocol for sensor data acquisition, which interacts with the MODBUS protocol to enable information transmission to SCADA systems. Tong W. et al. [26] designed a multi-protocol gateway supporting bidirectional conversion among Modbus RTU, ASCII, and TCP.

However, there is still a big gap in the research on the interoperability conversion between UDP transport and serial links and their reliability enhancement mechanisms. In particular, the research on how to maintain industrial-grade communication reliability under weak network conditions, such as high packet loss rate or out of order rate, has not been fully explored.

### 2.2. Temporal Properties

Currently, both domestic and international studies employ various performance metrics to evaluate fieldbus systems like Modbus, such as network utilization rate $P_{util}$, packet loss rate $P_{loss}$, network efficiency $P_{eff}$, and throughput $Q_{thr}$.

**Figure 2:**
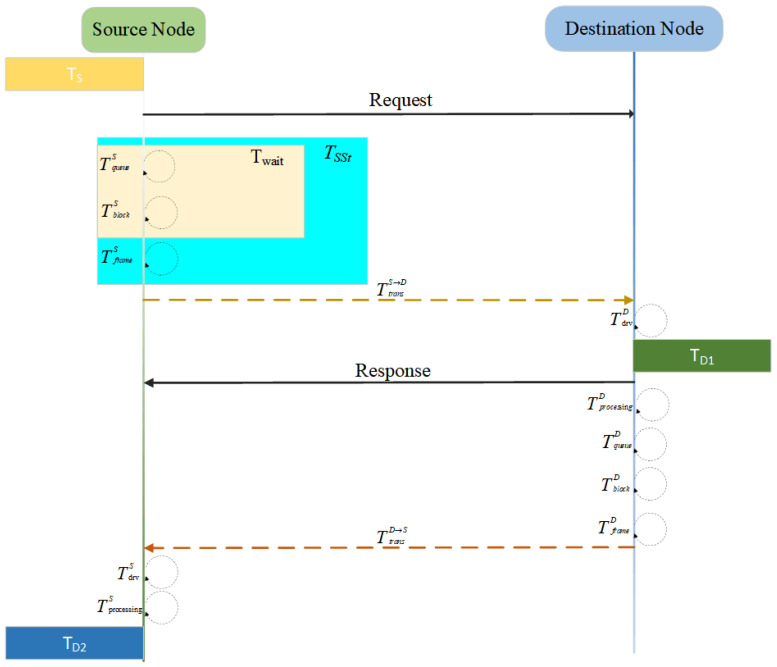
Transmission delay model.

The transmission delay $T_{delay}$ of a fieldbus message is defined as follows: when the source node starts to send a request frame at the timestamp $T_{S}$, the destination node receives the frame at $T_{D1}$ and immediately generates a response to send it after processing, and the source node ultimately receives the response successfully at $T_{D2}$, and the transmission delay $T_{delay}$ is the time interval from $T_{S}$ to $T_{D2}$. It mainly consists of node’s total transmission delay $T_{SSt}$, channel transmission delay $T_{trans}$, node reception delay $T_{drv}$, and application layer processing delay $T_{processing}$. Here, $T_{SSt}$ includes the waiting delay $T_{wait}$ and the frame transmission delay $T_{frame}$, where $T_{wait}$ comprises the queuing delay at source node $T_{queue}$ and blocking delay $T_{block}$ (which occurs when the transmission channel is occupied by other messages). The transmission delay model is illustrated in Figure 2 above (the upper table for each delay represents the corresponding node or direction).

Based on the transmission delay model listed in Figure 2, the following mathematical model can be listed:

(1) $$T_{\mathrm{delay}}=T_{D1}-T_{S}+T_{D2}-T_{D1}=T_{D2}-T_{S}=T_{\mathrm{SSt}}+T_{\mathrm{trans}}+T_{\mathrm{drv}}+T_{\mathrm{processing}}$$

In the above equation, $T_{\mathrm{SSt}}=T_{\mathrm{queue}}^{S}+T_{\mathrm{block}}^{S}+T_{\mathrm{frame}}^{S}+T_{\mathrm{queue}}^{D}+T_{\mathrm{block}}^{D}+T_{\mathrm{frame}}^{D},$ $T_{\mathrm{trans}}=T_{\mathrm{trans}}^{S\to D}+T_{\mathrm{trans}}^{D\to S},$ $T_{\mathrm{drv}}=T_{\mathrm{drv}}^{D}+T_{\mathrm{drv}}^{S},$ $T_{\mathrm{processing}}=T_{\mathrm{processing}}^{D}+T_{\mathrm{processing}}^{S}.$ The total delay in message transmission for *N* nodes of the system to complete a periodic communication is

(2) $$\sum_{i\in N_{\mathrm{nodes}}}\sum_{j=1}^{M(i)}T_{\mathrm{delay}}^{\left(i,j\right)}$$

Let *N* be the number of nodes, $N_{node}$ the node set, and M(i) the message count per node. Compared to Modbus TCP, Modbus UDP omits the three-way handshake and four-way termination, reducing the total transmission delay $T_{delay}^{eum}$ in periodic N-node communication. The elimination of redundant control frames increases throughput $Q_{thr}$ and network efficiency $P_{eff}$, while avoiding TCP state machine design accelerates node response and data processing.

The above analysis indicates that Modbus UDP exhibits lower latency than Modbus TCP, thereby achieving enhanced real-time capabilities in the Modbus UDP protocol.

### 2.3. Markov Chain

Evaluating the $P_{loss}$ and $P_{util}$ alone as common metrics for the performance analysis of communication systems is based on static and independent event assumptions, ignoring temporal and spatial correlations. The application of a Markov chain can model the dynamic channel state and can show the temporal correlation and state transfer characteristics of the communication process, so this study analyzes the data transmission process through a Markov chain.

A Markov chain is a discrete stochastic process possessing the Markov property [27]. Formally, given a probability space (Ω, *F*, *P*) and a sequence of random variables $X=\{X_{n}:n>0\}$ indexed by a countable set, where each state $X_{n}=S_{i}$ belongs to state space *S*, the Markov property requires the conditional probability satisfies

(3) $$P\left(X_{t+1}|X_{t},\ldots ,X_{1}\right)=P\left(X_{t+1}|X_{t}\right)$$

Then *X* is called a Markov chain.

In a Markov chain, the change in the state of a random variable over time steps is referred to as state transition. The state transition probability, which is the most direct and crucial value reflecting the properties of the Markov chain, is defined as

(4) $$P_{jk}\left(m\right)=P\left\{X_{m+1}=k|X_{m}=j\right\}$$

The term $P_{jk}\left(m\right)$ represents the one-step transition probability of a system transitioning from state *j* at time step *m* to state *k* at the next time step. In stochastic processes, these probabilities are typically organized into a matrix form. Assuming the system has *n* + 1 possible states, the transition matrix *P* is constructed by arranging all state transition probabilities as

(5) $$P_{n,n+1}\equiv \left(p_{i_{n}i_{n+1}}\right)=\left[\begin{array}{ccc} P_{0,0} & \ldots & P_{0,n}\\ \vdots & \ddots & \vdots \\ P_{n,0} & \ldots & P_{n,n} \end{array}\right]$$

For an irreducible and positive recurrent Markov chain, there exists a unique stationary distribution $\pi =(\pi_{0},\pi_{1},\ldots ,\pi_{n})$ [28], satisfying

(6) $$\left\{\begin{array}{c} \pi P=\pi ,\\ \sum_{i=1}^{n}\pi \left(i\right)=1 \end{array}\right.$$

Here, π(i) represents the probability that the system is in state *i* under steady-state conditions. The first equation πP=π indicates that the steady-state distribution π is the left eigenvector of the state transition probability matrix *P* corresponding to the eigenvalue 1. This condition ensures the conservation of the probability distribution: once the system reaches a steady state, the probability distribution remains unchanged after any single transition step. The second equation $\sum_{i=0}^{n}\pi \left(i\right)=1$ imposes a normalization constraint on the probability distribution, guaranteeing that the sum of probabilities over all possible states equals 1. Furthermore, if the Markov chain satisfies irreducibility (all states are mutually reachable) and positive recurrence (the expected return time to any state is finite), the steady-state distribution π exists uniquely. The analytical solution for π can then be obtained by solving the system of linear equations.

In recent years, Markov chains have found extensive applications across diverse domains. Giovanni Sansavini et al. [29] developed a discrete hidden Markov mathematical model and an exponentially weighted moving average (EWMA) model to analyze and predict time delays in Wide-Area Power Systems (WAPS). These predicted delays were then fed into a predictor to mitigate the performance degradation of Load Frequency Control (LFC). Bishop J. [30] applied the Markov chain to the driving cycles. Fei Z. [31] investigated the reachable set synthesis for nonlinear delayed hidden semi-Markov jump systems with actuator saturation and multiple cyber-attacks. This study will employ Markov chains to analyze the transmission of Modbus data.

In this study, a Modbus AUDP and Modbus RTU protocol conversion experimental gateway is designed based on STM32 and W5500, and the reliability and real-time performance of Modbus data transmission based on the experimental gateway are investigated and analyzed by introducing a Markov chain.

## 3. System Design and Implementation

### 3.1. Overall Architecture

**Figure 3:**
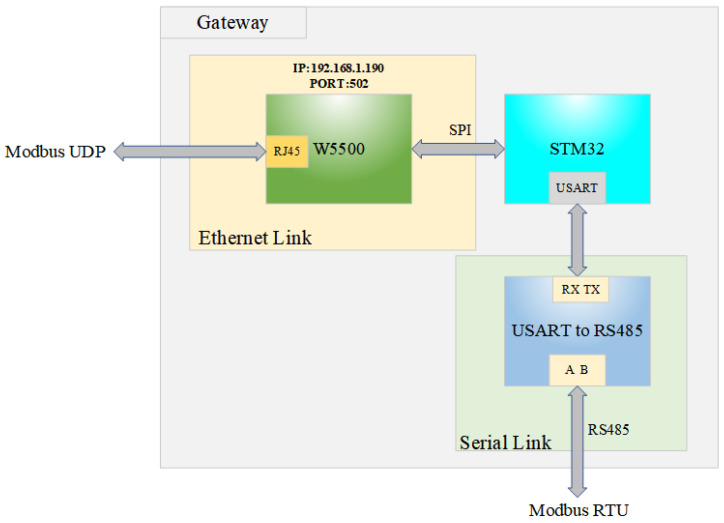
System overall architecture diagram.

Figure 3 shows the overall architecture of the system, which is mainly composed of a W5500 Ethernet controller, STM32 microcontroller, and USART to RS485 modules.

The W5500 serves as the core for Ethernet access, integrating a 10/100 M PHY and a hardware-based TCP/IP stack that supports IPv4, TCP, UDP, Internet Control Message Protocol (ICMP), Address Resolution Protocol (ARP), and Point-to-Point Protocol over Ethernet (PPPoE). It incorporates 32 KB of dual-port SRAM for transceiver buffering and manages up to 8 independent hardware sockets simultaneously. Utilizing an Serial Peripheral Interface (SPI) bus with speeds up to 80 MHz, its low-power design features a 1.8 V core voltage and operating current below 120 mA, meeting the stringent energy efficiency requirements of industrial IoT edge devices. W5500 is bound to the designated port through the pre-configured IP address, listens to and accepts Modbus UDP data messages from the host computer, and transfers the messages to STM32 for data processing through SPI communication after the Modbus UDP messages are received. After the Modbus UDP message is received, the message is transmitted to STM32 for data processing through SPI communication, which converts the Modbus UDP message into Modbus RTU message, and then sends the Modbus RTU message to the USART to RS485 module through USART to convert the serial signal into differential signal for RS485 transmission, and then finally transmits the data to the related equipment through RS485 bus. After receiving the data, the related device generates a Modbus RTU response frame and transmits it back to the gateway through RS485 bus, and finally converts the Modbus RTU message into Modbus UDP message and sends it back to the host computer through the signal conversion, data processing, and network connection of the three modules of the gateway.

### 3.2. Hardware Design

**Figure 4:**
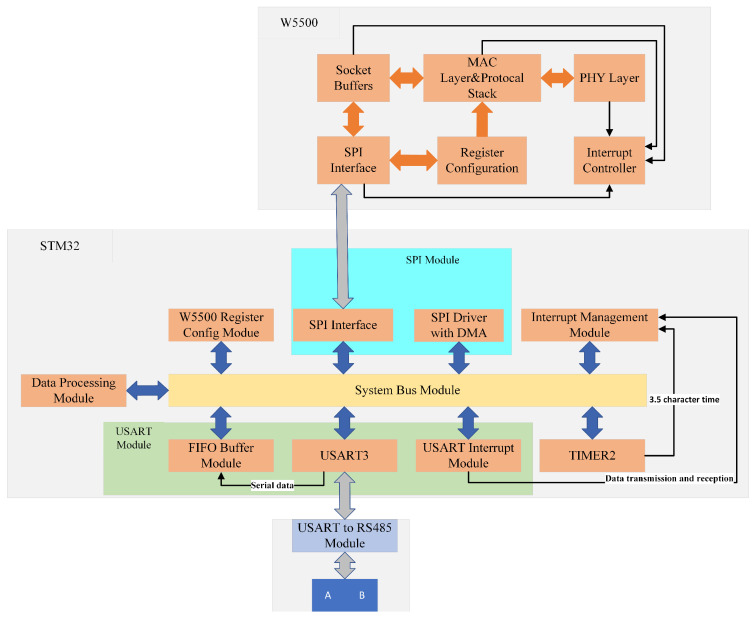
Hardware architecture diagram.

Figure 4 shows the hardware architecture diagram, which includes a USART to RS485 conversion module, USART module, timer module, SPI interface module, SPI driver with DMA module, W5500 register configuration module, and data-processing module.

- USART to RS48 Module: This is used to convert between USART signals and RS485 bus differential signals to realize the transmission between STM32 microcontroller and RS485 bus.
- USART Module: This is composed of a software FIFO buffer, USART3 peripheral, and interrupt service mechanism. The data is captured in real-time via receive completion interrupt and stored in the FIFO. Frame termination is determined using idle interrupt. The main loop asynchronously reads FIFO data for protocol parsing/processing, enabling transceiver status monitoring and data flow scheduling.
- TIMER2: This utilizes TIM2 to configure the auto-reload value (ARR) and prescaler (PSC) to generate timeout interrupts. It detects the end of a Modbus RTU frame transmission by measuring a 3.5-character time interval.
- SPI Module: It comprises a SPI interface (Mode 0, 8-bit frame @18 MHz) and SPI-DMA driver with double buffering. It enables zero-CPU-overhead full-duplex data transfer between STM32 and W5500 through cyclic DMA channel configuration.
- W5500 Register Configuration Module: The physical layer (PHY) configuration and interrupt enable of W5500 is realized through the configuration of W5500-related registers by STM32.
- Data-Processing Module: It performs CRC-16 validation and bidirectional Modbus AUDP/Modbus RTU protocol conversion.
- Interrupt Management Module: It handles centralized interrupt events (e.g., USART idle, SPI transfer complete, and W5500 status) with priority-based response logic via NVIC, ensuring the timely clearing of interrupt flags through hardware-level register operations.

#### 3.2.1. STM32 Minimum System Schematic

**Figure 5:**
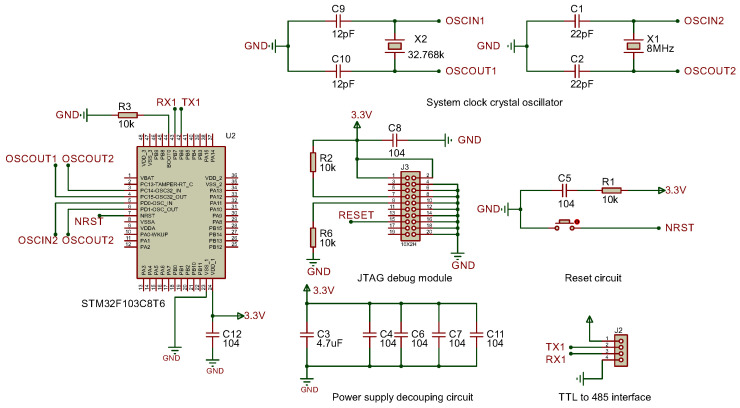
STM32 minimum system.

The system adopts the STM32F103C8T6 microcontroller as the core of the gateway, and its minimum system schematic is shown in Figure 5. The system operates at 3.3 V and adopts a dual clock design: the 8 MHz main crystal provides the system clock reference, and the 32.768 kHz low-speed crystal drives the real-time clock (RTC). Both oscillator circuits are stabilized using matched capacitors to optimize performance. A push-button reset circuit ensures reliable reboot, and serial communication is realized through a USART interface, which is connected to a TTL-to-RS485 module for signal conversion. A standard JTAG debugging interface compatible with the J-Link debugger and the Keil MDK development environment is integrated for firmware programming, real-time monitoring, and debugging.

**Figure 6:**
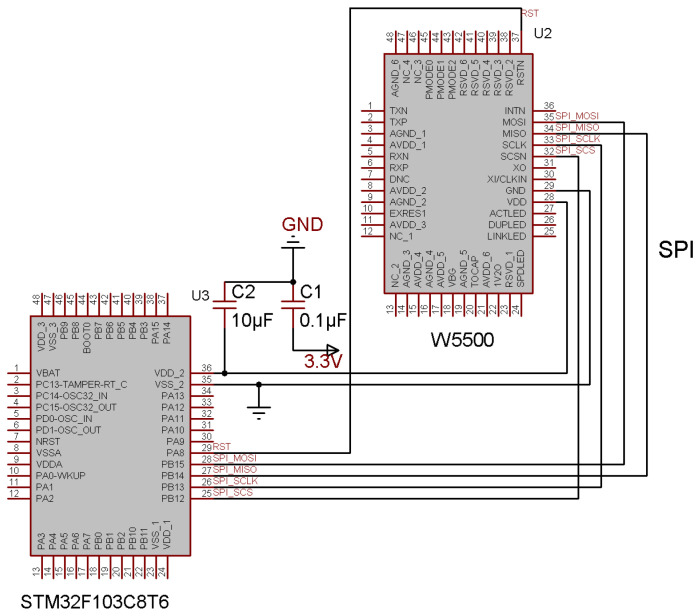
SPI communication.

The W5500 Ethernet controller communicates with the STM32 microcontroller via SPI. As shown in Figure 6, the STM32F103C8T6’s PB12 (SCS), PB13 (SCLK), PB14 (MISO), and PB15 (MOSI) pins are directly connected to the corresponding SPI pins of the W5500, enabling seamless data exchange.

#### 3.2.2. Power Supply Module

**Figure 7:**
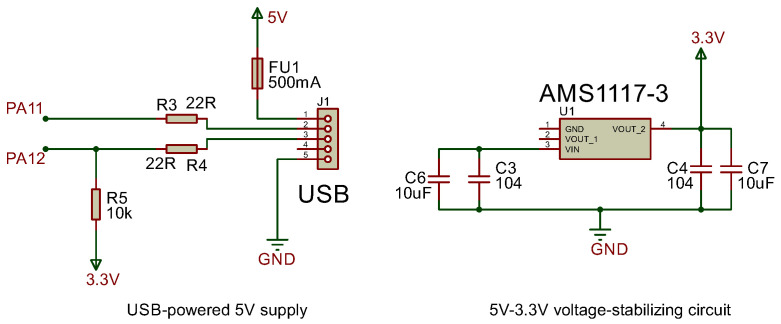
Power supply module circuit.

The USART to RS485 module operates on a 5 V power supply, incorporating a USB-powered 5 V source. For the STM32 microcontroller requiring 3.3 V operation, a dedicated 5 V to 3.3 V voltage regulator circuit is implemented to ensure stable 3.3 V output (see power supply module schematic in Figure 7).

#### 3.2.3. Interface Conversion Module

**Figure 8:**
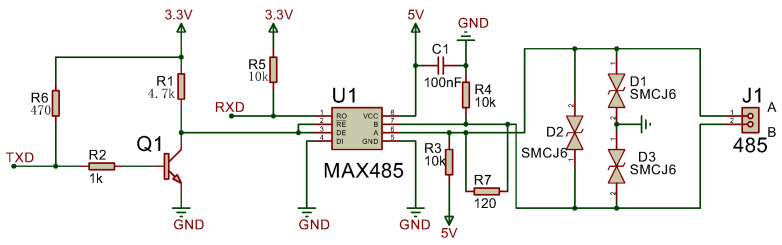
USART to RS485 circuit.

The interface conversion module enables conversion between USART signals and RS485 differential signals, with automatic transmit/receive state switching. The USART to RS485 circuit (shown in Figure 8) employs the MAX485 chip as the converter, utilizing an NPN transistor to switch transmit/receive states. During data reception, TXD remains at a high level, turning the transistor on. This pulls the RE pin of the MAX485 to a low level (enabled), allowing the RO pin to output received RS485 bus data to the microcontroller. During transmission, TXD outputs a low level, turning the transistor off. The DE pin is then driven high to enable the transmit mode.

### 3.3. Software Design

**Figure 9:**
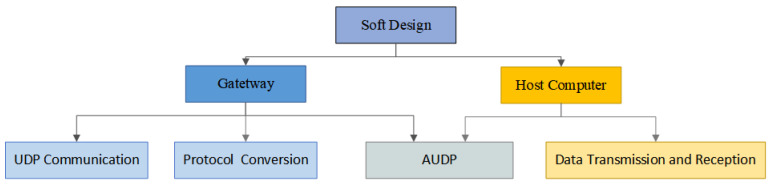
Software architecture diagram.

The software design is divided into two modules: gateway and host computer. The gateway module is divided into the establishment of UDP communication, the design of protocol conversion, and the realization of AUDP mechanism. The host computer module is divided into data transmission and reception (via socket programming), and the realization of the AUDP mechanism. Figure 9 shows the software design framework.

#### 3.3.1. UDP Communication

**Figure 10:**
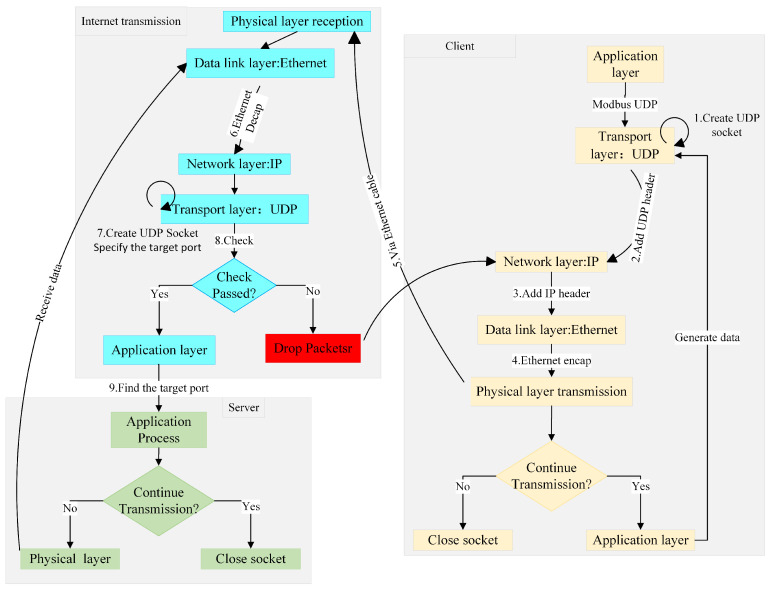
UDP communication workflow.

The Modbus UDP transport layer operates on UDP. As shown in Figure 10, the UDP communication workflow begins with both the client and server creating sockets via the socket() system call. The server then binds to a specific port using bind() to enable persistent listening and initialize network parameters such as the local IP address and port. The client is dynamically assigned a temporary port by the system. After generating data at the application layer, the client sends packets using sendto(), which triggers the transport layer to append a UDP header, the network layer to encapsulate the destination IP, and the data link layer to construct Ethernet frames (including MAC addresses) before transmission via the physical layer. Upon receiving data, the server decapsulates each layer sequentially, validates the data, and uses the source port in the UDP header to identify the sender and the destination port to route packets correctly. The server then calls recvfrom() to receive the validated data. Finally, both parties close the sockets via close() to release allocated ports and kernel resources.

#### 3.3.2. Implementation of the AUDP Mechanism

To enhance the reliability of UDP-based communication, this study introduces four reliability mechanisms through an in-depth analysis of UDP transmission characteristics: (1) CRC checksum mechanism, (2) retransmission mechanism, (3) Transaction ID matching mechanism, and (4) exponential backoff mechanism.

- Analysis of UDP Validation Mechanism

UDP employs its own checksum mechanism covering the IP pseudo-header, UDP header, and data fields using a 16-bit 1’s complement summation algorithm, with an undetected error probability denoted as $P_{UDP}^{undetected}$:

(7) $$P_{\mathrm{undetected}}^{\mathrm{UDP}}=\frac{1}{2^{16}}\approx 1.53\times 10^{-5}$$

In the high-noise environment, where the channel bit error rate reaches up to $10^{-4}$, relying solely on the UDP transport layer’s checksum mechanism severely weakens the error detection capability. Numerous corrupted packets caused by channel interference may go undetected and reach the application layer directly, ultimately triggering unpredictable anomalies in the receiver’s business logic. However, with the addition of a CRC-16 checksum at the application layer, the total undetected probability becomes $P_{undetected}^{undetected}$:

(8) $$P_{\mathrm{undetected}}^{\mathrm{total}}=P_{\mathrm{undetected}}^{\mathrm{UDP}}\times P_{\mathrm{undetected}}^{\mathrm{CRC}}=\frac{1}{2^{16}}\times \frac{1}{2^{16}}=\frac{1}{2^{32}}\approx 2.33\times 10^{-10}$$

The undetected error probability has been significantly reduced.

**Figure 11:**
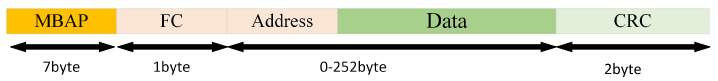
Modbus UDP frame structure.

Therefore, this study implements dual-layer validation at both the transport and application layers by appending a two-byte CRC-16 checksum to the end of the Modbus UDP frame as illustrated in Figure 11. After the data is transmitted to the gateway, the received CRC checksum (denoted as ’receive_CRC’) is extracted from the Modbus UDP frame. A new CRC calculation is performed on the data to derive the ’caculate_CRC’. If ’receive_CRC’ matches ’caculate_CRC’, this confirms that the data integrity has been preserved during transmission.

- Analysis of Retransmission Mechanism

A Markov chain model is constructed for the UDP-based transmission process, where $S_{1}$, $S_{2}$, and $S_{3}$ denote the waiting-for-response state, success state, and failure state, respectively. The corresponding state transition probability matrix *P* is defined as

(9) $$P=\left[\begin{array}{ccc} 0 & 1-\beta & \beta \\ 1 & 0 & 0\\ \gamma & 0 & 1-\gamma \end{array}\right]$$

where β is the probability that the request is waiting for a timeout, and γ is the probability that the failure state triggers a retransmission. After the retransmission is introduced, $S_{2}$ retransmits the request with probability γ and maintains the failure state with 1 − γ. According to Equation (6), the stationary distribution $\left(\pi_{1},\pi_{2},\pi_{3}\right)$ satisfies $\left(\pi_{1},\pi_{2},\pi_{3}\right)P=\left(\pi_{1},\pi_{2},\pi_{3}\right)$, where $\pi_{2}$ represents the long-term probability of the system being in the success state, and its value is provided in Equation (10):

(10) $$\pi_{2}=\frac{\gamma (1-\beta )}{\beta +\gamma }$$

**Figure 12:**
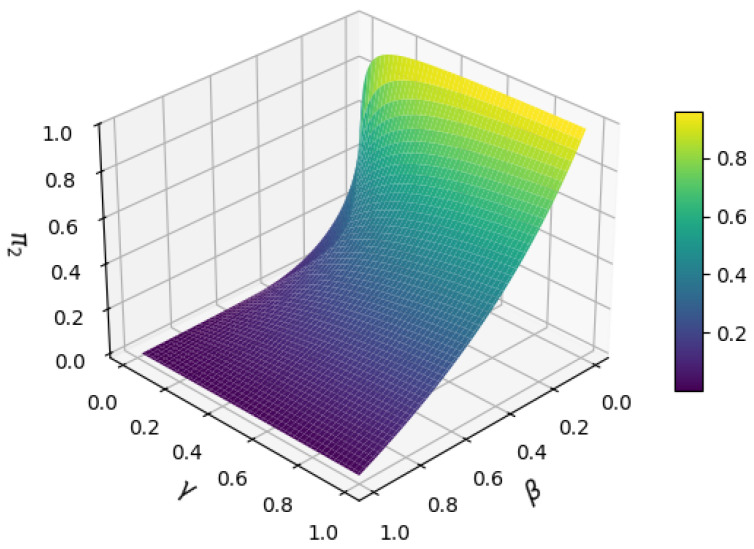
Surface plot of the effect of β and γ on $\pi_{2}$.

Figure 12 illustrates the variation of steady-state success rate $\pi_{2}$ with timeout probability β and retransmission probability γ. When β→0 and γ→1, $\pi_{2}$ approaches 1; conversely, when β→1 or γ→0, $\pi_{2}$ decreases significantly. This demonstrates that the retransmission mechanism is essential to ensure reliability.

Consequently, this study implements a retransmission mechanism at the application layer of UDP to enhance the reliability of Modbus UDP transmissions.

- Analysis of Transaction ID Matching Mechanism

Assuming that the transmission process conforms to the Markov property, the following three assumptions are made: (1) After each transmission, the system waits for a response with a timeout period set to *T* seconds. (2) The network exhibits random delays, and the response arrival time follows a probability density function f(t), and f(t) follows an exponential distribution. (3) The network has a $P_{loss}$ rate *p*, where each transmission request is lost with probability *p*, necessitating retransmission.

A logical error occurs when the delayed response of request *n* arrives after the timeout period and is incorrectly matched to the subsequent request n+1. The probability that the response delay to request *n* exceeds *T* is

(11) $$P_{\mathrm{delay}}=\int_{T}^{\infty }f\left(t\right)\;dt$$

If the response to request *n* exceeds the timeout period *T*, and request n+1 is transmitted within the time window [T,T+δt], the response *n* may be incorrectly attributed to request n+1. Assuming the request interval is a fixed period τ, the probability of erroneous matching is given by

(12) $$P_{\mathrm{error}}=P_{\mathrm{delay}}\cdot \frac{\Delta t}{\tau }\overset{\Delta t∼U(T,T+\tau )}{\approx }\frac{1}{2}P_{\mathrm{delay}}$$

Assume each request can be retransmitted up to *N* times with a retransmission interval of *T* seconds, where retransmitted requests carry identical payloads to the original request but lack Transaction ID. The response to the *k*-th retransmission may be erroneously matched to any subsequent request by the error-prone mechanism. Let $P_{\mathrm{delay}}$ denote the probability that the response delay exceeds *T* for both the original request and each retransmission. The total erroneous matching probability $P_{\mathrm{error}\_\mathrm{total}}$ is then given by

(13) $$P_{\mathrm{error}\_\mathrm{total}}=\sum_{k=0}^{N}P_{\mathrm{delay}}^{k+1}\cdot \frac{1}{2}=\frac{1}{2}P_{\mathrm{delay}}\frac{1-P_{\mathrm{delay}}^{R+1}}{1-P_{\mathrm{delay}}}$$

When Transaction IDs are implemented, the correct matching criteria are defined as follows: (1) Each request is assigned a unique Transaction ID, and a response must carry the matching Transaction ID to be accepted. (2) Let the bit length of the Transaction ID be *b*, then the ID space size is *R* = $2_{b}$. (3) Erroneous matching occurs only if the response Transaction ID coincidentally matches the currently pending request ID.

Assuming the ID collision probability caused by malicious attacks or coincidental matches is $\frac{1}{R}$, the error probability per individual request becomes

(14) $$P_{\mathrm{error}\_\mathrm{id}}=\frac{1}{R}$$

For *N* retransmissions, the total error probability is expressed as

(15) $$P_{\mathrm{error}\_\mathrm{total}}=\sum_{k=0}^{N}P_{\mathrm{delay}}^{k+1}\cdot \frac{1}{2}=\frac{1}{2}P_{\mathrm{delay}}\frac{1-P_{\mathrm{delay}}^{R+1}}{1-P_{\mathrm{delay}}}$$

When $\frac{1}{R}\ll 1$, Equation (17) can be approximated using the Taylor expansion formula:

(16) $$P_{\mathrm{error}\_\mathrm{total}\_\mathrm{id}}\approx \frac{N+1}{R}$$

For *b* = 16 and *R* = $2^{16}$, assuming ($P_{delay}$ = 0.1), substituting the parameters into Equations (13) and (16) yields a total erroneous matching probability of $P_{\mathrm{error}\_\mathrm{total}}\approx 5.55\%$ without Transaction ID, while the implementation of Transaction ID reduces this probability to $P_{\mathrm{error}\_\mathrm{total}\_\mathrm{id}}\approx 0.0061\%$. The significant disparity between $P_{\mathrm{error}\_\mathrm{total}}$ and $P_{\mathrm{error}\_\mathrm{total}\_\mathrm{id}}$ ($P_{\mathrm{error}\_\mathrm{total}}\gg P_{\mathrm{error}\_\mathrm{total}\_\mathrm{id}}$) demonstrates that embedding a Transaction ID matching mechanism at the application layer over UDP is essential to enhance transmission reliability, even with timeout-based retransmissions already in place.

According to the above derivation, a 16-bit Transaction ID is assigned to each packet to match the request and response, and the request sent by the upper computer is resent if it does not receive the uplink message from the slave device within the specified time to prevent the loss of the request frame. Referring to the Transaction ID verification mechanism of Modbus TCP, the MBAP of Modbus TCP is transplanted to Modbus UDP (as shown in Figure 11), the request and response matching mechanism is added to Modbus UDP at the application layer, an incremental Transaction ID is generated before each request to ensure the uniqueness of the request, and the request is sent to the gateway. When sending to the gateway, the gateway will record the Transaction ID, and then append it to the Modbus UDP response frame. When the host computer parses the response, it will extract the Transaction ID and verify it. If the request frame is consistent with the Transaction ID in the response frame, it means that it is a valid response; otherwise, it enters the retransmission mechanism.

- Analysis of Transaction ID Matching Mechanism

To reduce the network load and increase the probability of retransmission, the program waits for a period before each retransmission, with an initial waiting time ‘base_delay’, and doubles the waiting time after each retry.

**Figure 13:**
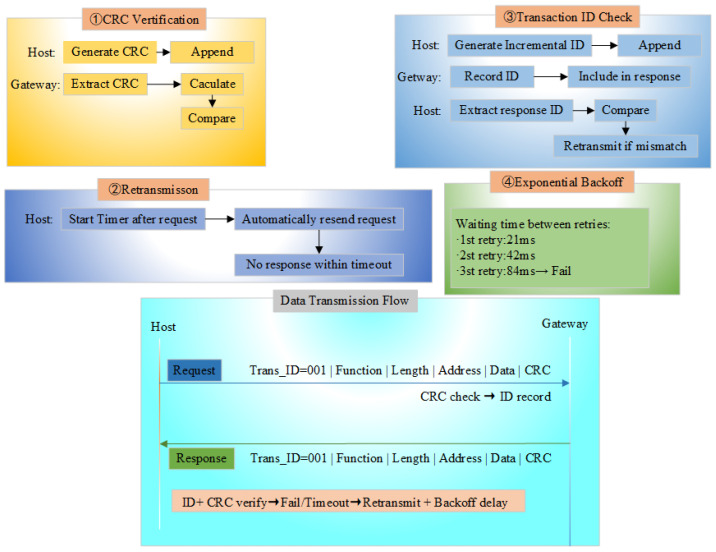
Schematic diagram of the Modbus AUDP reliability mechanism.

By integrating the analysis of the UDP validation mechanism, retransmission mechanism, Transaction ID matching mechanism, and exponential backoff mechanism, and to reduce the computational load on the gateway, the reliability mechanism is implemented through collaboration between the host computer and the gateway. Figure 13 shows the schematic diagram of the Modbus AUDP reliability mechanism.

#### 3.3.3. Protocol Conversion Design

**Figure 14:**
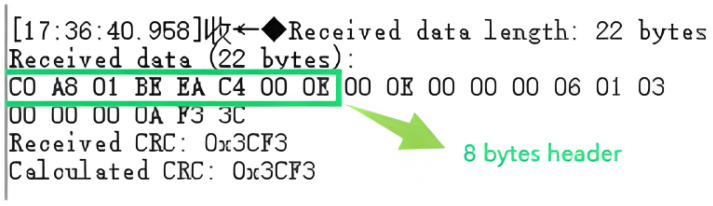
UDP data received by W5500.

For the conversion from Modbus AUDP to Modbus RTU, after the W5500 Ethernet controller receives Modbus AUDP data, it stores the data into the data reception buffer RX_Buffer in the STM32 via SPI communication. For UDP communication based on the W5500 Ethernet controller, the 8-byte header Figure 14 must be skipped before protocol conversion to enable data decapsulation and encapsulation. Upon entering the formal conversion process, the first step is to ensure the received data length is at least 18 bytes (8 bytes UDP header + 7 bytes MBAP header + at least 1 byte PDU + 2 bytes CRC). If the length requirement is met, retain the CRC from the received Modbus UDP frame, perform CRC validation on the remaining data, and compare the result with the retained CRC. If they match, proceed to the next step. Subsequently, extract the MBAP header (bytes 9 to 15), record the Transaction ID from the MBAP header, verify whether the protocol identifier (bytes 11–12) is 0, extract the Unit ID (byte 15) as the Modbus RTU address code, copy the remaining bytes after the Unit ID, and finally append a CRC to complete the conversion from Modbus AUDP to Modbus RTU. The converted data is then transmitted via serial port to the downstream device.

**Figure 15:**
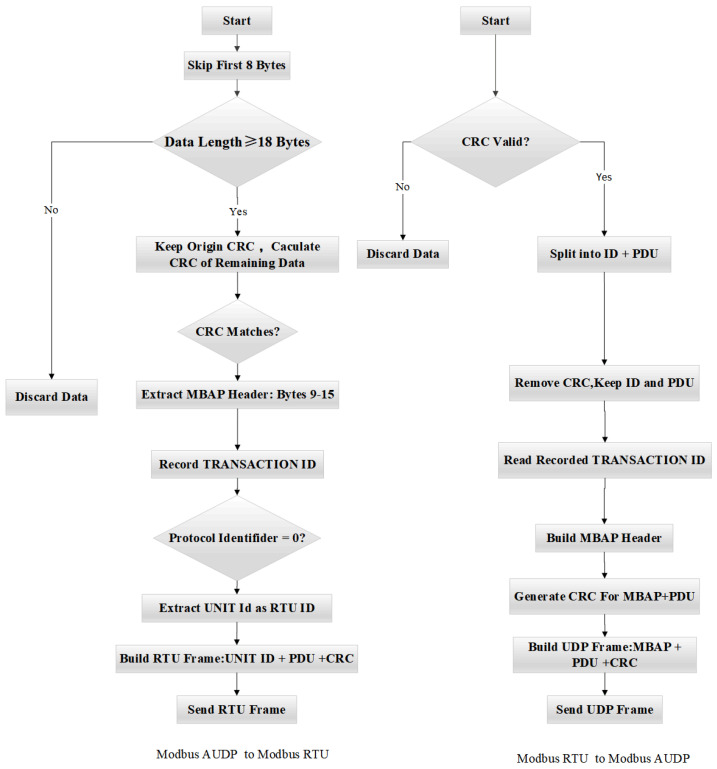
Gateway data-processing flowchart.

**Figure 16:**
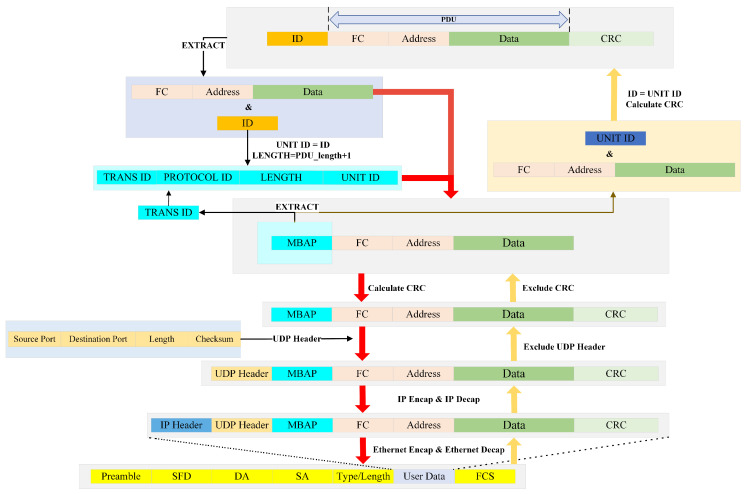
Data encapsulation flowchart.

For the conversion from Modbus RTU to Modbus AUDP, after the gateway receives the RTU response frame via the serial port, it performs CRC validation to verify data integrity. The RTU response frame is then decomposed into Slave Address + PDU + CRC Code. The CRC is removed, retaining the Slave ID and PDU. The Slave Address and the previously recorded Transaction ID are used to construct the MBAP header of the Modbus UDP. The PDU portion of the RTU frame is appended to the MBAP header, followed by a CRC calculation on the combined MBAP+PDU data. The resulting CRC is appended to form the complete Modbus UDP frame. This completes the conversion from Modbus RTU to Modbus AUDP, and the data is transmitted back to the upper-level device via the RJ45 Ethernet port. The gateway’s data-processing flowchart and frame encapsulation flowchart are shown in Figure 15 and Figure 16, respectively.

#### 3.3.4. Implementation of the Host-Side Software

**Figure 17:**
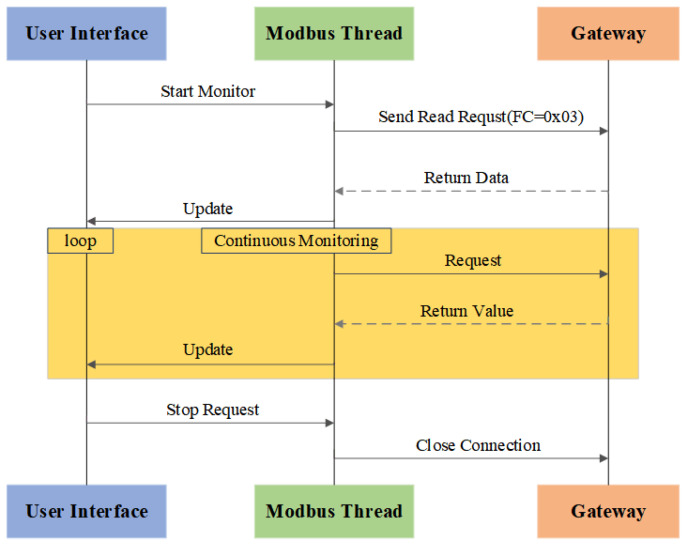
Flowchart of the host computer program.

Python 3.12.0 boasts mature Modbus open-source libraries (e.g., pymodbus) that can be directly utilized for Modbus communication implementation, significantly reducing the development cycle. Furthermore, Python 3.12.0 ’s rich graphics libraries (e.g., Matplotlib and Tkinter) facilitate user interface design, while its scientific computing ecosystem (e.g., NumPy and Pandas) supports efficient integration with data-processing tools. Consequently, the Modbus data transmission, reception, and processing modules on the host computer are implemented using Python 3.12.0. Figure 17 shows the flowchart of the host computer program. The host sends read register requests to the gateway, and the device returns current data values upon response. The system continuously cycles through the ’send request–receive response–update interface’ process. The user interface, implemented in Python 3.12.0 and running on the host computer, updates and displays the retrieved contents of holding registers in real-time. For example, when the host computer sends a Modbus read holding register request via Python 3.12.0, the slave device receives the request, generates a response frame, and transmits it back to the host, after which the interface dynamically reflects the acquired data. When monitoring is manually stopped, the system safely terminates communication threads and closes the network interfaces to release resources.

## 4. Experimental Results and Analysis

**Figure 18:**
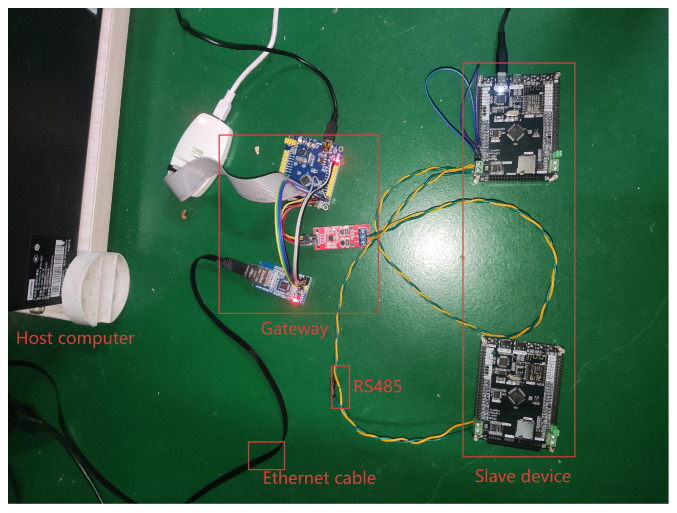
Photograph of the designed gateway prototype used for experimental testing.

This section establishes a Request–Response Latency mathematical model and derives the Average Latency delay model using mathematical expectation. Experimental analysis based on both models demonstrates the following: (1) A comparative analysis of the Request–Response Latency between network-side TCP and UDP transmissions reveals comparable real-time performance under non-degraded network conditions, while TCP exhibits significantly higher latency than UDP in weak network conditions. (2) In-depth analysis of Modbus AUDP data transfer processes under weak network conditions, leveraging the Average Latency model and Markov Chain, determines the optimal value of $N_{max}$. (3) Comparative analysis of AUDP and UDP transmissions under weak network conditions provides systematic validation of Modbus AUDP’s real-time responsiveness and transmission reliability. Figure 18 is the photograph of the designed gateway prototype used for experimental testing.

### 4.1. Request–Response Latency and Average Latency Modeling

Request–Response Latency is defined as the total time from the transmission of a request frame to the completion of response reception, and its components are shown in Equation (17):

(17) $$\mathrm{Request}-\mathrm{Response Latency}=RTT+t_{\mathrm{processing}}+T_{\mathrm{out}}\times N$$

where RTT (Round-Trip Time) and $t_{processing}$ represent the round-trip channel latency and application-layer processing time during the final successful retransmission, respectively. $T_{out}$ denotes the timeout period: if the host fails to receive a response within $T_{out}$, the retransmission mechanism is triggered, with *N* being the number of retransmission failures.

During data frame transmission, the randomness of packet loss and retransmission mechanisms leads to probabilistic variations in actual latency across frames. To analyze the overall system performance, expectation must be introduced to model the Average Latency. The Average Latency is defined as the mean value of the total time from sending a request frame to fully receiving its response, which includes the Round-Trip Time (RTT), application processing time $t_{processing}$, and $T_{out}$. Let *k* be the number of retransmissions required before successful transmission, and let *p* represent the probability of transmission failure. The probability of success at the k+1 attempt equals the product of the failure probabilities of the first *k* retransmissions and the success probability of the (k+1)-th retransmission. The expected number of Retransmissions is derived as

(18) $$E\left(K\right)=\sum_{k=1}^{N_{\max}}k\times p^{k}\left(1-p\right)$$

Each retransmission introduces a timeout period, and the expectation E[K] represents the average retransmission count. Therefore, by defining $D_{avg}$ as the Average Latency, the Average Latency can be expressed as

(19) $$D_{\mathrm{avg}}=RTT+t_{\mathrm{processing}}+T_{\mathrm{out}}\times E\left(K\right)=RTT+t_{\mathrm{processing}}+T_{\mathrm{out}}\times \sum_{k=1}^{N_{\max}}k\times p^{k}\left(1-p\right)$$

### 4.2. Comparison of TCP-Based and UDP-Based Transmission on the Network-Side

To conduct real-time analysis of Modbus UDP and Modbus TCP, data transmission/reception tests were performed for both protocols under ideal conditions (0% packet loss rate and 0% out-of-order rate) and weak network conditions (30% $P_{loss}$ rate and 10% out-of-order rate simulated by the weak network simulation tool Clumsy). Latency is measured through Python and Wireshark packet capture: while Python sends requests and receives responses, Wireshark captures the packets; then the Modbus packets captured by Wireshark are filtered out and exported in ‘csv’ format; and finally the exported ’csv’ file is imported into Python to calculate the latency.

**Figure 19:**
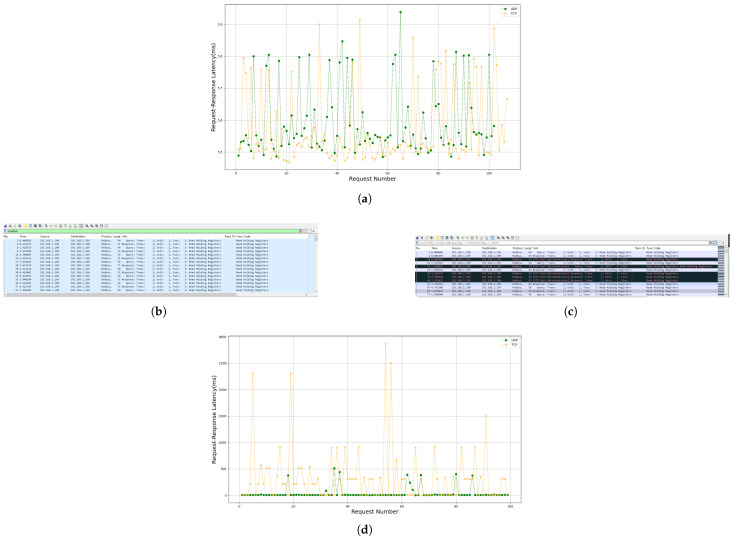
(**a**) Comparison of Request–Response Latency between UDP-based and TCP-based transmissions under 0% packet loss and 0% out-of-order conditions. (**b**) Packet capture diagram of UDP-based transmission under weak network conditions. (**c**) Packet capture diagram of TCP-based transmission under weak network conditions. (**d**) Comparison of Request–Response Latency between UDP-based and TCP-based transmissions under weak network conditions.

Figure 19a shows the Request–Response Latency comparison between 100 Modbus UDP and Modbus TCP requests sent to the slave device under ideal conditions. Figure 19b,c display the packet capture results of 100 Modbus TCP and Modbus UDP requests sent to the gateway under weak network conditions, while Figure 19d compares their Request–Response Latency.

Figure 19a reveals that under ideal conditions, the Request–Response Latency of TCP-based and UDP-based transmissions is nearly identical, with a difference below 1 ms. Figure 19b–d demonstrate that under weak network conditions, UDP-based transmission exhibits smaller latency fluctuations, whereas TCP-based transmission suffers from frequent congestion control mechanism triggers. This results in the Request–Response Latency of Modbus UDP transmissions remaining stable at approximately 5 ms, while the latency for Modbus TCP packet transmissions exhibits significant instability.

Therefore, Modbus UDP demonstrates significant advantages over Modbus TCP in real-time performance under weak network conditions.

### 4.3. Modbus AUDP Gateway Analysis

**Figure 20:**
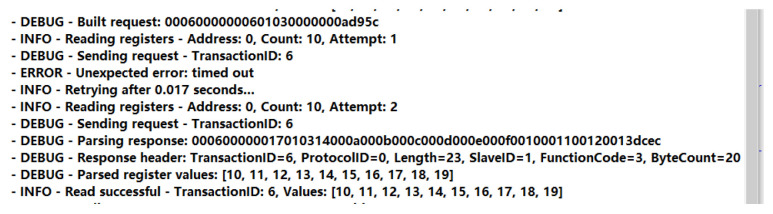
Data transmission and reception diagram.

As illustrated in Figure 20, the transmission and reception results of Modbus AUDP data under weak network conditions demonstrate that the gateway successfully processed the data. The sender appended a CRC checksum ‘d95c’ to the Modbus UDP request frame, while the gateway generated a corresponding checksum ‘dcec’ for the response frame, implementing dual-layer validation at both the transport and application layers. Furthermore, the application layer ensured data integrity through a Transaction ID matching mechanism: each request was assigned a unique Transaction ID, and responses were validated by matching the Transaction ID during parsing. Upon receiving a response, the system extracted and compared the Transaction ID; only when the ID matched was the response deemed valid, triggering data updates. Retransmission mechanisms were activated immediately upon initial request timeout, dynamically adjusting retry intervals using an exponential backoff algorithm to mitigate network congestion. As shown in the figure, the request successfully retrieved data during the second retransmission interval.

**Figure 21:**
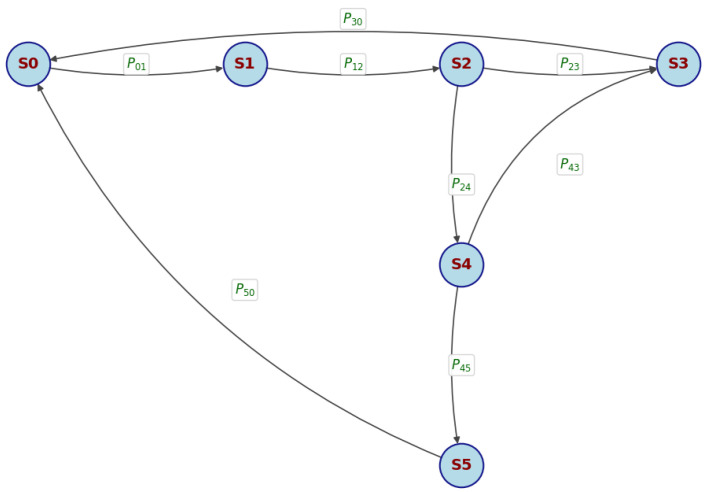
State transition diagram.

For the Modbus data transmission process under AUDP, let $S_{0}$ denote the waiting to send state, $S_{1}$ the packet sending state, $S_{2}$ the waiting for response state, $S_{3}$ the successful reception state, $S_{4}$ the retransmission state, and $S_{5}$ the communication failure state. The state transitions are illustrated in Figure 21:

Assume each retransmission is independent, with identical success probabilities and no differentiation between retransmission attempts. As shown in Figure 21, any state can transition to another within finite steps, and each state returns to itself in finite expected time. Thus, the system is irreducible and positive recurrent, implying the existence of steady-state probabilities analyzable via Markov chains. Based on Figure 21, the state transition matrix can be constructed as follows:

(20) $$P=\left[\begin{array}{cccccc} 0 & P_{01} & 0 & 0 & 0 & 0\\ 0 & 0 & P_{12} & 0 & 0 & 0\\ 0 & 0 & 0 & P_{23} & P_{24} & 0\\ P_{30} & 0 & 0 & 0 & P_{43} & 0\\ 0 & 0 & 0 & P_{43} & 0 & P_{45}\\ P_{50} & 0 & 0 & 0 & 0 & 0 \end{array}\right]$$

Substituting the state transition matrix P into the general balance condition Equation (6), the steady-state balance equations are derived as follows:

(21) $$\left\{\begin{array}{c} \pi_{0}=\pi_{3}P_{30}+\pi_{5}P_{50}\\ \pi_{1}=\pi_{0}P_{01}\\ \pi_{2}=\pi_{1}P_{12}\\ \pi_{3}=\pi_{2}P_{23}+\pi_{4}P_{43}\\ \pi_{4}=\pi_{2}P_{24},\\ \pi_{5}=\pi_{4}P_{45}\\ \sum_{i=0}^{5}\pi_{i}=1 \end{array}\right.$$

The maximum retransmission count $N_{max}$ directly affects the system’s steady-state probabilities. In a network environment with a 20% $P_{loss}$ rate, experiments were conducted by transmitting 1000 data frames under different $N_{max}$ values. From Figure 18, $P_{01}$ = $P_{12}$ = $P_{30}$ = $P_{50}$ = 1, $P_{43}$ = 1 −$P_{45}$, $P_{23}$ =1 −$P_{24}$. Simplify the above equation to obtain Equation (22):

(22) $$\left\{\begin{array}{c} \pi_{0}=\frac{1}{4+P_{24}}\\ \pi_{1}=\pi_{2}=\pi_{0}\\ \pi_{3}=\pi_{0}\left(1-P_{24}P_{45}\right)\\ \pi_{4}=\pi_{0}P_{24}\\ \pi_{5}=\pi_{0}P_{24}P_{45}\\ \sum_{i=0}^{5}\pi_{i}=1 \end{array}\right.$$

The steady-state probabilities are determined by the probability of entering retransmission ($P_{24}$) and the probability of retransmission failure ($P_{45}$) under a packet loss rate of 20% (the experimental values of $P_{24}$ and $P_{45}$ are listed in Table 1). The steady-state probabilities of each state corresponding to each $N_{max}$ are statistically analyzed, with the results summarized in Table 1:

As detailed in Table 1, the steady-state probabilities $\pi_{0}$, $\pi_{1}$, $\pi_{2}$, and $\pi_{3}$—corresponding to the core communication phases of waiting to send, packet sending, waiting for response, and successful reception—remain nearly constant (23.5–23.6%) as $N_{\max}$ increases. This stability indicates that, under the given network conditions, the system spends the majority of its operational time cycling through these essential states, with their combined probability consistently exceeding 93.8%. Such a distribution reflects the protocol’s efficiency and the effectiveness of its retransmission mechanism in maintaining normal operation. The probability of being in the retransmission state ($\pi_{4}$) remains below 6.1% across all tested $N_{\max}$ values, suggesting that retransmissions are infrequent and well-managed, thus preventing excessive network congestion. Most notably, the probability of entering the communication failure state ($\pi_{5}$) decreases sharply as $N_{\max}$ increases, dropping from 0.36% at $N_{\max}=1$ to just 0.006% at $N_{\max}=6$. This trend demonstrates the significant benefit of allowing more retransmission attempts, as it greatly reduces the likelihood of unrecoverable failures. However, as derived in Equation (19), it is important to note that while increasing $N_{\max}$ enhances reliability, it may also lead to increased average transmission delay. Therefore, a practical upper limit of $N_{\max}=6$ is adopted to balance reliability and latency. In summary, the steady-state analysis in Table 1 quantitatively confirms that the proposed AUDP-based transmission system achieves high reliability and efficiency under weak network conditions, with the vast majority of time spent in controllable states.

To further determine the optimal $N_{MAX}$, this study employs the entropy weight method to evaluate $N_{MAX}\in \left[1,6\right]$ through composite scoring. The parameters $P_{24}$, $P_{45}$, $\pi_{4}$, and $\pi_{5}$ are selected as negative indicators, while $\pi_{3}$ (successful reception probability) and $\pi_{0}$ + $\pi_{1}$ + $\pi_{2}$ + $\pi_{3}$ (cumulative probability of essential operational states) serve as positive indicators.

First, normalize the positive and negative indicators:

(23) $$\left\{\begin{array}{cc} X_{ij}^{\mathrm{norm}}=\frac{\max\left(X_{j}\right)-X_{ij}}{\max\left(X_{j}\right)-\min\left(X_{j}\right)} & \mathrm{Negative}\;\mathrm{Indicator}\\ X_{ij}^{\mathrm{norm}}=\frac{X_{ij}-\min\left(X_{j}\right)}{\max\left(X_{j}\right)-\min\left(X_{j}\right)} & \mathrm{Positive}\;\mathrm{Indicator} \end{array}\right.$$

where $X_{j}$ and $X_{ij}$ represent the value of indicator *j* in Table 1 and the value of indicator *j* when $N_{MAX}$ = *i*, respectively.

Next, calculate the weight values using the following formula:

(24) $$\left\{\begin{array}{c} f_{ij}=\frac{x_{ij}^{\mathrm{norm}}}{\sum_{i=1}^{m}x_{ij}^{\mathrm{norm}}}\\ e_{j}=-\frac{1}{\ln n}\sum_{i=1}^{m}f_{ij}\ln f_{ij}\\ d_{j}=1-e_{j}\\ w_{j}=\frac{d_{j}}{\sum_{j=1}^{n}\left(1-e_{j}\right)} \end{array}\right.$$

The optimal $N_{MAX}$ is determined through three phases.

$e_{ij}$ and $f_{ij}$ denote the information entropy and proportion of the *j*-th indicator in Table 1, $d_{j}$ is the differentiation coefficient, *n* is the sample count, and $w_{j}$ is the weight of the *j*-th indicator. Take the negative indicator $P_{24}$ as an example:

(1) Normalize it: Substitute into Equation (23) the values of item $P_{24}$ under different $N_{\max}$ in Table 2, where $\max(x_{j})=25.6\%$ and $\min(x_{j})=23.5\%$, and $x_{ij}$ is the value to be normalized. The calculated normalized results for item $P_{24}$ are
  $$X_{i,P_{24}}^{\mathrm{norm}}=\left\{0.4286,\;1.0000,\;0.8571,\;0.3333,\;0.3809,\;0\right\}.$$
(2) From (1), obtain the information entropy $e_{j}$ of item $P_{24}$ and the proportion $f_{ij}$ of $P_{24}$ at different $N_{\max}$; the results are
  $$f_{ij}=\left\{0.1429,\;0.3333,\;0.2857,\;0.1111,\;0.1270,\;0.0001\right\},$$
  so
  $$e_{j}=-\frac{1}{\ln n}\sum f_{ij}\ln f_{ij}=0.8532.$$
(3) According to Equation (21),
  $$d_{j}=1-e_{j}=0.1468,\;w_{j}=\frac{d_{j}}{\sum_{j=1}^{n}\left(1-e_{j}\right)}=20.55\%.$$
  The same applies for $P_{45}$, $\pi_{3}$, $\pi_{4}$, $\pi_{5}$, and $\pi_{0}+\pi_{1}+\pi_{2}+\pi_{3}$, and the calculation results are given in Table 2.

Finally, compute the comprehensive score for each $N_{max}$ via weighted summation using the following formula:

(25) $$S_{i}=\sum w_{j}\times x_{ij}^{\mathrm{norm}}$$

From Table 3, the highest score is obtained when A = 2. Therefore, the maximum retransmission count in the gateway application layer is set to $N_{max}$ = 2.

### 4.4. Network-Side Comparison of AUDP-Based and UDP/TCP-Based Transmission

**Figure 22:**
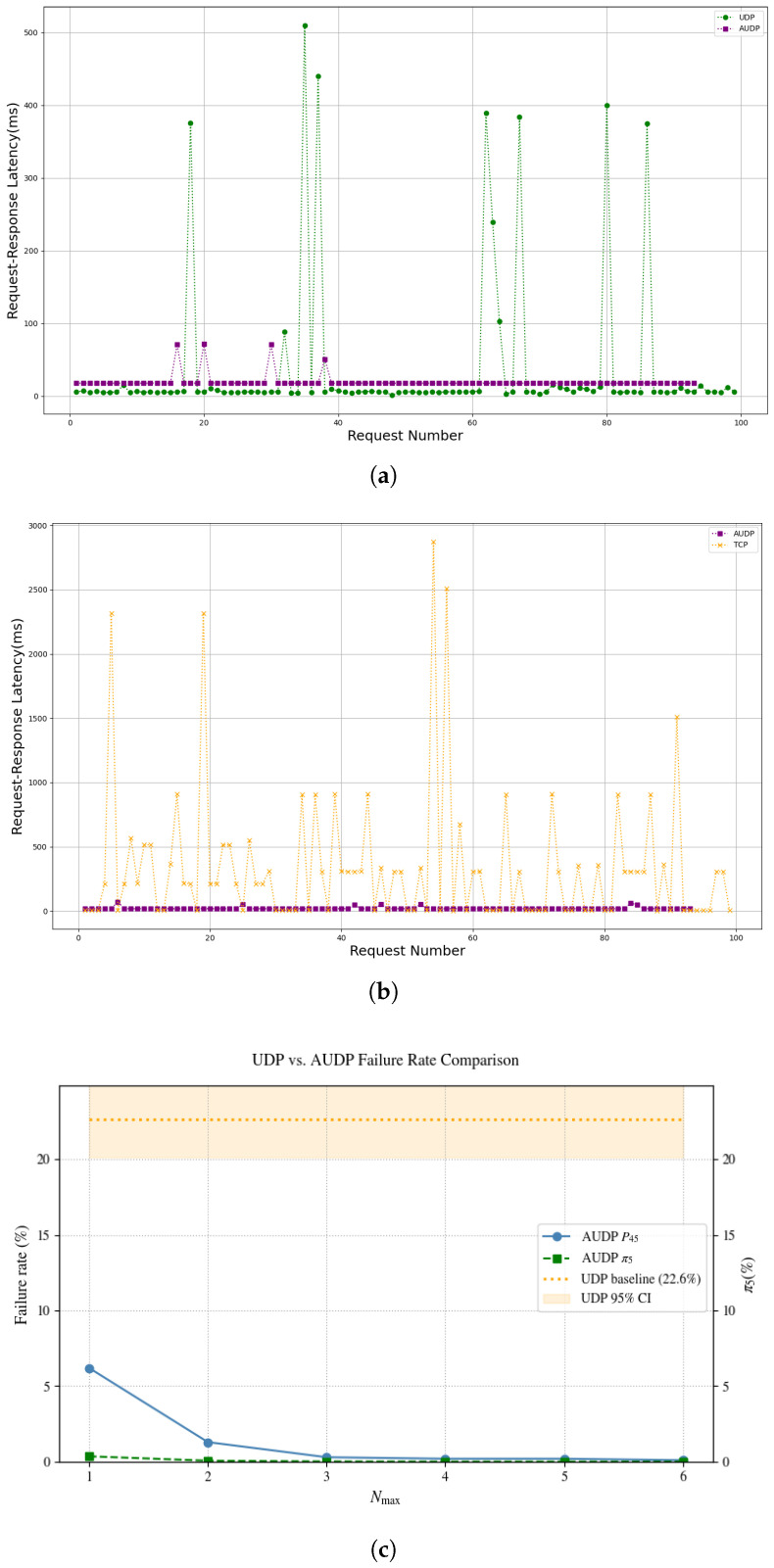
(**a**) Comparison of Request–Response Latency between UDP-based and AUDP-based transmissions under weak network conditions. (**b**) Comparison of Request–Response Latency between TCP-based and AUDP-based transmissions under weak network conditions. (**c**) Failure rate comparison of Modbus UDP and AUDP gateways with 20% $P_{loss}$.

After integrating reliability mechanisms into the application layer of Modbus UDP (Modbus AUDP) with the retransmission count $N_{max}$ set to 2, a $P_{loss}$ rate of 30%, and a out-of-order rate of 10%, the Request–Response Latency slightly increased to approximately 17 ms as shown in Figure 22a. Despite this increase, Modbus AUDP’s latency remains significantly lower than that of TCP-based transmission (Figure 22b).

Under a reduced $P_{loss}$ of 20% ($N_{max}$ = 2, no out-of-order rate), 1000 Modbus UDP request were transmitted. As shown in Figure 22c, the communication failure rate of Modbus UDP gateway is 22.6% (95% CI: 20.1–25.3%), whereas that of Modbus AUDP gateway decreases to 0.3% ($P_{45}$) at $N_{\max}=3$, and the steady-state probability of $S_{5}$ also remains at a very low level. The failure rate of Modbus AUDP gaeway is consistently below the lower bound of Modbus UDP gateway’s confidence interval (CI), indicating a significant improvement in reliability.

## 5. Conclusions

The Modbus AUDP-RTU gateway proposed in this study significantly reduces the communication delay and effectively solves the problem of the low communication reliability of UDP. The modeling analysis of UDP transmission based on Markov chain provides theoretical support for system performance optimization, and the experimental results show that the success rate of data transmission after applying AUDP is increased to more than 98% compared with 77.4% of Modbus UDP-RTU, and the real-time performance is also significantly improved compared with Modbus TCP-RTU, which proves the advantages of the AUDP mechanism in terms of reliability and real-time performance. The proposed Modbus AUDP-RTU gateway offers significant practical value for IoT applications, enabling the efficient and reliable real-time monitoring and control of equipment in real-world factory settings. This solution not only helps enterprises reduce upgrade costs and improve operational efficiency but also lays a solid foundation for the large-scale adoption of Industry 4.0 technologies.

Future research will focus on optimizing the AUDP retransmission mechanism and refining state transition models to achieve adaptive retransmission capabilities. This includes dynamically adjusting maximum retransmission attempts based on network conditions while expanding the gateway’s protocol support to align with Industry 4.0 and IoT standards. In particular, the integration of OPC UA will be considered to enable semantic interoperability and secure machine-to-machine communication through its information modeling and encryption features, while the adoption of MQTT will facilitate lightweight publish–subscribe messaging, optimize bandwidth usage, and support efficient cloud-edge synchronization in IoT environments. These enhancements are expected to bridge legacy Modbus systems with modern industrial frameworks, thereby improving both real-time reliability and cross-platform scalability. In addition, we will also explore the introduction of a Modbus multi-master mechanism to integrate a multi-master coordination framework, enabling orderly concurrency of multiple services while maintaining compatibility with existing Modbus devices.

## Figures and Tables

**Table 1:** Statistical table of steady-state probabilities.

$N_{\max}$	$P_{24}$	$P_{45}$	$\pi_{0}=\pi_{1}=\pi_{2}$	$\pi_{3}$	$\pi_{4}$	$\pi_{5}$	$\pi_{0}+\pi_{1}+\pi_{2}+\pi_{3}$
1	24.7%	6.2%	23.55%	23.19%	5.82%	0.36%	93.84%
2	23.5%	1.3%	23.62%	23.54%	5.55%	0.07%	94.4%
3	23.8%	0.3%	23.61%	23.58%	5.64%	0.02%	94.39%
4	24.9%	0.2%	23.55%	23.53%	5.82%	0.01%	94.18%
5	24.8%	0.2%	23.55%	23.53%	5.85%	0.01%	94.18%
6	25.6%	0.1%	23.50%	23.49%	6.02%	0.006%	93.99%

**Table 2:** Calculation results of key parameters.

Configuration	$e_{j}$	$d_{j}$	$w_{j}$
NMMS_$p_{24}$	0.8532	0.1468	20.55%
NMMS_$p_{45}$	0.9030	0.0970	13.57%
NMMS_$\pi_{3}$	0.9032	0.0968	13.55%
NMMS_$\pi_{4}$	0.8602	0.1380	19.30%
NMMS_$\pi_{5}$	0.9033	0.0967	13.53%
NMMS_$\pi_{0}+\pi_{1}+\pi_{2}+\pi_{3}$	0.8607	0.1393	19.50%

**Table 3:** Comprehensive scores and rankings based on the entropy weight method.

$N_{\max}$	Comprehensive Score ($S_{i}$)	Ranking
1	0.1802	6
2	0.9462	1
3	0.9319	2
4	0.6559	3
5	0.6633	4
6	0.4374	5

## Data Availability

The data in this paper is obtained from real control experiments. Because the laboratory test data is not publicly available, if there is a need for them, contact the corresponding author.
